# Local Treatment and Treatment-Related Adverse Effects Among Patients With Advanced Prostate Cancer

**DOI:** 10.1001/jamanetworkopen.2023.48057

**Published:** 2023-12-18

**Authors:** Saira Khan, Su-Hsin Chang, Mei Wang, Eric H. Kim, Martin W. Schoen, Carleena Rocuskie-Marker, Bettina F. Drake

**Affiliations:** 1 Research Service, St Louis Veterans Affairs Medical Center, St Louis, Missouri; 2 Division of Public Health Sciences, Department of Surgery, Washington University in St Louis School of Medicine, St Louis, Missouri; 3 Division of Urologic Surgery, Department of Surgery, Washington University in St Louis School of Medicine, St Louis, Missouri; 4 Department of Internal Medicine, Saint Louis University School of Medicine, St Louis, Missouri; 5 Immunology and Microbial Pathogenies Program, West Virginia University, Morgantown

## Abstract

**Importance:**

Recent data suggest that local treatment with radical prostatectomy or radiation may improve survival outcomes in men with advanced prostate cancer. However, evidence is lacking on treatment-related adverse effects among men with advanced prostate cancer.

**Objective:**

To assess the association of local treatment on treatment-related adverse effects among men diagnosed with advanced prostate cancer.

**Design, Setting, and Participants:**

This cohort study assessed men diagnosed with advanced prostate cancer (defined as T4, N1, and/or M1 prostate cancer) between January 1, 1999, and December 31, 2013, with follow-up through December 31, 2021, who were treated at Veterans Health Administration medical centers.

**Exposure:**

Local treatment with radical prostatectomy or radiation.

**Main Outcomes and Measures:**

Main outcomes were treatment-related adverse effects, including constitutional, gastrointestinal, pain, sexual function, and urinary function conditions, at 3 intervals after initial treatment (≤1 year, >1 to ≤2 years, and >2 to ≤5 years) after initial treatment.

**Results:**

This cohort study consisted of 5502 men (mean [SD] age, 68.7 [10.3] years) diagnosed with advanced prostate cancer. Of the cohort, 1705 men (31.0%) received local treatment. There was a high prevalence of adverse conditions in men receiving both local and nonlocal treatment, and these adverse conditions persisted for more than 2 years to 5 years or less after initial treatment. A total of 916 men (75.2%) with initial local treatment and 897 men (67.1%) with initial nonlocal treatment reported the presence of at least 1 adverse condition for more than 2 years to 5 years or less after initial treatment. In the first year, local treatment (vs nonlocal) was associated with adverse gastrointestinal (multivariable-adjusted odds ratio [AOR], 4.08; 95% CI, 3.06-5.45), pain (AOR, 1.57; 95% CI, 1.35-1.83), sexual (AOR, 2.96; 95% CI, 2.42-3.62), and urinary (AOR, 2.25; 95% CI, 1.90-2.66) conditions. Local treatment (without secondary treatment) remained significantly associated with adverse gastrointestinal (AOR, 2.39; 95% CI, 1.52-3.77), sexual (AOR, 3.36; 95% CI, 2.56-4.41), and urinary (AOR, 1.39; 95% CI, 1.09-1.78) conditions at more than 2 years to 5 years or less after treatment.

**Conclusions and Relevance:**

In this cohort study of men with advanced prostate cancer, local treatment was associated with persistent treatment-related adverse effects across multiple domains. These results suggest that patients and clinicians should consider the adverse effects of local treatment when making treatment decisions in the setting of advanced prostate cancer.

## Key Points

**Question:**

What is the burden of treatment-related adverse effects among men receiving local treatment with radical prostatectomy or radiation for advanced prostate cancer?

**Findings:**

In this retrospective cohort study of 5502 US veterans diagnosed with advanced prostate cancer, local (vs nonlocal) treatment was associated with adverse gastrointestinal, sexual, and urinary conditions within the year after initial treatment and remained significantly associated with these adverse conditions more than 2 years to 5 years or less after treatment.

**Meaning:**

Patients and clinicians should consider the adverse effects of local treatment when making treatment decisions in the setting of advanced prostate cancer.

## Teaser

This cohort study of veterans with advanced prostate cancer assesses the prevalence of treatment-related adverse effects and their association with local vs nonlocal treatment.

## Introduction

Although the overall survival rate of prostate cancer (PCa) is high, among those diagnosed with metastatic disease, the 5-year survival rate is only 32%.^1^ The standard of care for men with advanced PCa is androgen deprivation therapy (ADT). However, the role of local therapy, including radical prostatectomy (RP)^2^ and radiation,^3^ has been increasingly considered, and increasing evidence suggests that local therapy may also provide a survival benefit in advanced disease.

Previous research has shown that local treatment improves overall and PCa-specific survival among men with advanced PCa.^2,4,5,6^ Moreover, local treatment can improve survival in veterans with advanced PCa.^7^ Although existing studies have established the importance of analyzing quality of life in men with newly diagnosed or localized PCa when considering local treatment options,^8,9,10,11^ evidence among men with advanced disease remains limited.

This cohort study aims to define the added morbidity of local treatment, across multiple domains, to patients with advanced PCa, particularly considering that the added survival benefit of local therapy may be small.^3,12,13^ Ongoing clinical trials may provide further insights into quality of life in men with advanced disease^14^; however, to our knowledge, this is one of the first studies to look at local treatment and adverse effects in men with advanced PCa across multiple domains, including constitutional, gastrointestinal, pain, sexual, and urinary conditions, up to 5 years after treatment. Currently, 2 ongoing clinical trials^15,16^ are comparing local therapy with systematic therapy alone. The results of this study will be valuable, both now and after the results of these trials are published, as clinicians and patients weigh the relative harms and benefits of local therapy in the advanced setting. Using data from the nationwide Veterans Health Administration (VHA), this study assessed (1) the prevalence of treatment-related adverse effects among patients with advanced PCa and (2) the association between the receipt of local treatment and treatment-related adverse effects across 3 periods.

## Methods

### Study Population

The data for this cohort study were derived from the Veterans Affairs Central Cancer Registry (VACCR) within the VHA system. The VACCR contains data on cancer type, stage, grade, treatment, and race. Race is reported because it is a strong risk factor PCa outcomes and an important confounder to consider. Other clinical variables, including laboratory values and other diagnoses of interest, were obtained from the Veterans Affairs Corporate Data Warehouse. Using the VACCR, we identified a retrospective cohort of US veterans who were diagnosed with PCa between January 1, 1999, and December 31, 2013 (n = 149 821), and followed up through December 31, 2021. We included veterans diagnosed with advanced disease, which was comprehensively defined as T4, N1, and/or M1 (T4N1M1) PCa (n = 5718). After excluding 216 patients with missing treatment, our analytic cohort included 5502 men (eFigure 1 in Supplement 1). This study received approval from the St Louis Veterans Affairs Medical Center, Washington University, and US Department of Defense institutional review boards. A waiver of informed consent was received from these institutional review boards because the use or disclosure of the requested information involves no more than a minimal risk to the privacy of individuals. This study followed the Strengthening the Reporting of Observational Studies in Epidemiology (STROBE) reporting guideline.

### Outcomes

Outcomes in this study were treatment-related adverse effects at 3 intervals in the following categories: (1) constitutional, including hot flashes; (2) gastrointestinal, including diarrhea and proctitis; (3) chronic pain; (4) sexual, including erectile dysfunction; and (5) urinary, including incontinence, cystitis, and overactive bladder. All conditions were identified by *International Classification of Diseases, Ninth Revision, Clinical Modification* (*ICD-9-CM*) or *International Statistical Classification of Diseases, Tenth Revision, Clinical Modification* (*ICD-10-CM*) codes, *Current Procedural Terminology* codes, and/or the medication used to treat these conditions (eTable 1 in Supplement 1).

Intervals were defined based on time from initial treatment (ie, by definition, all participants had initial treatment at baseline). For radiation, intervals were defined from time of first radiation treatment. The following 3 intervals were used: 1 year or less, more than 1 year to 2 years or less, and more than 2 to 5 years or less after initial treatment.

### Exposure

Our exposure was receipt of local treatment, defined as RP, radiation, or RP with radiation. Nonlocal treatment was defined as hormone therapy only, chemotherapy only, or a combination of both (ie, systemic therapy). To account for any secondary treatment that occurred between 1 and 5 years after initial treatment, our exposure was further stratified by local treatment first with no secondary treatment, local treatment first followed by any secondary treatment, and only nonlocal treatment (referent). Treatment that occurred within 1 year of initial treatment was considered adjuvant and not counted as secondary treatment.

### Covariates

Covariates included the following: age at diagnosis (<50, 50 to <60, 60 to <70, or ≥70 years), race (Black, White, or other [including Asian, Native American, and Pacific Islander]), body mass index (BMI [calculated as weight in kilograms divided by height in meters squared]) at diagnosis (<18.5, 18.5-<25, 25-<30, or ≥30), clinical stage (I, II, III, or IV), grade (1, 2, 3, or 4), family history of malignancy (yes vs no), rurality (rural vs urban), and whether the treating hospital is an academic center (academic vs nonacademic). Grade was defined as well differentiated (grade 1), moderately differentiated (grade 2), poorly differentiated (grade 3), and undifferentiated (grade 4). An unknown category was created for variables with missing data. Covariates were selected based on PCa risk factors and known confounders from the literature.

### Statistical Analysis

We computed proportions of each category for the included demographic and clinical characteristics, stratified by local treatment status, in the study sample. We also constructed comparisons between local and nonlocal treatments among patients with each condition across the 3 intervals. For each outcome category in each interval, multivariable logistic regression was used to estimate the adjusted odds ratios (AORs) for local treatment groups (compared with nonlocal treatment). Models were adjusted for age, race, BMI, family history of cancer, rurality, and treatment at an academic center. All tests were 2-sided. Statistical significance was defined as α = .05. Analyses were performed using SAS software, version 9.2 (SAS Institute Inc).

### Sensitivity Analyses

We conducted several sensitivity analyses. First, we ran models with additional adjustment for prostate-specific antigen. Second, we ran subgroup analyses among men receiving surgery vs radiation. Third, to examine a more restrictive definition of advanced disease, we used an alternate definition of advanced disease, defined as T4 or M1 (T4/M1) PCa (n = 3438). Finally, in another sensitivity analysis, to compare adverse effects across the 3 intervals following the first treatment, we restricted the cohort to patients who had at least 5 years of follow-up (n = 2554 for T4/N1/M1 and n = 1250 for T4/M1).

## Results

### Cohort Characteristics

This cohort study consisted of 5502 men (mean [SD] age, 68.7 [10.3] years; 1566 [28.5%] Black, 3778 [68.8%] White, 29 [0.5%] other [including Asian, Native American, and Pacific Islander], and 129 [2.3%] unknown). A total of 3964 [72.0%] of men were urban residents, 1866 (33.9%) had node-positive disease, 4424 (80.4%) had stage 4 disease, and 3942 (71.6%) had grade 3 disease (Table 1). A total of 1705 patients (31.0%) received local treatment as their initial treatment vs 3797 (69.0%) who did not. The local treatment group was different from the nonlocal treatment group across age, BMI category, residential location, cancer stage and grade, and family history of reportable cancer. Among those receiving local treatment, 943 (55.3%) received RP, 667 (39.1%) received radiation, and 95 (5.6%) received both.

**Table 1.** Characteristics of Men Diagnosed With Prostate Cancer From 1997 to 2013^a^

Characteristic	Men with metastatic prostate cancer (T4N1 M1), No. (%)		
	Nonlocal treatment (n = 3797)	Local treatment (n = 1705)	Total (N = 5502)
Age group, y			
<50	35 (0.9)	51 (3.0)	86 (1.6)
50 to <60	517 (13.6)	485 (28.5)	1002 (18.2)
60 to <70	1137 (29.9)	837 (49.1)	1974 (35.9)
≥70	2108 (55.5)	332 (19.5)	2440 (44.3)
Race^b^			
Black	1090 (28.7)	476 (27.9)	1566 (28.5)
White	2599 (68.5)	1179 (69.2)	3778 (68.8)
Other^c^	22 (0.6)	7 (0.4)	29 (0.5)
Unknown	86 (2.3)	43 (2.5)	129 (2.3)
BMI			
<18.5	101 (2.7)	21 (1.2)	122 (2.2)
18.5-<25	1262 (33.2)	447 (26.2)	1709 (31.1)
25-<30	1429 (37.6)	677 (39.7)	2106 (38.3)
≥30	1005 (26.5)	560 (32.8)	1565 (28.4)
Node positivity			
Yes	1483 (39.1)	383 (22.5)	1866 (33.9)
No	2314 (60.9)	1322 (77.5)	3636 (66.1)
Stage			
Missing	69 (1.8)	156 (9.2)	225 (4.1)
I	1 (0.0)	10 (0.6)	11 (0.2)
II	59 (1.6)	744 (43.6)	803 (14.6)
III	7 (0.2)	32 (1.9)	39 (0.7)
IV	3661 (96.4)	763 (44.8)	4424 (80.4)
Grade^d^			
Missing	539 (14.2)	95 (5.6)	634 (11.5)
1	18 (0.5)	6 (0.4)	24 (0.4)
2	454 (12.0)	271 (15.9)	725 (13.2)
3	2650 (69.8)	1292 (75.8)	3942 (71.6)
4	136 (3.6)	41 (2.4)	177 (3.2)
Location			
Urban	2767 (72.9)	1197 (70.2)	3964 (72.0)
Rural	900 (23.7)	485 (28.5)	1385 (25.2)
Unknown	130 (3.4)	23 (1.4)	153 (2.8)
Academic status			
Academic	1076 (28.3)	511 (30.0)	1587 (28.8)
Nonacademic	775 (20.4)	333 (19.5)	1108 (20.1)
Unknown	1946 (51.3)	861 (50.5)	2807 (51.0)
Family history of malignancy			
Yes	1068 (28.1)	640 (37.5)	1708 (31.0)
No	1545 (40.7)	696 (40.8)	2241 (40.7)
Unknown	1184 (31.2)	369 (21.6)	1553 (28.2)
Treatment type			
Radical prostatectomy	0	943 (55.3)	943(17.1)
Radiation	0	667 (39.1)	667 (12.1)
Adjuvant surgery and radiation^e^	0	95 (5.6)	95 (1.7)
Hormone only	3716 (97.9)	0	3716 (67.5)
Chemotherapy	81 (2.1)	0	81 (1.5)

Abbreviation: BMI, body mass index (calculated as weight in kilograms divided by height in meters squared).

^a^ Data are from the Veterans Affairs Central Cancer Registry.

^b^ Race was determined from medical records.

^c^ Other included Asian, Native American, and Pacific Islander.

^d^ Grade 1 indicates well differentiated; grade 2, moderately differentiated; grade 3, poorly differentiated; and grade 4, undifferentiated.

^e^ Defined as adjuvant if within 1 year of initial treatment.

### Burden of Treatment-Related Adverse Effects

Panel A in the Figure shows the prevalence of the treatment-related adverse effects at 3 time points after initial local or nonlocal treatment. A total of 916 men (75.2%) with initial local treatment and 897 men (67.1%) with initial nonlocal treatment reported the presence of at least 1 adverse condition for more than 2 years to 5 years or less after initial treatment. At 1 year or less after initial treatment, the prevalence of gastrointestinal (629 [8.8%] vs 150 [3.1%]), pain (1020 [59.8%] vs 1442 [38.0%]), sexual (629 [36.9%] vs 308 [8.1%]), and urinary (792 [46.5%] vs 689 [18.2%]), conditions was higher in men who received local treatment compared with men who received nonlocal treatment. The prevalence of constitutional conditions was similar across both treatment groups (623 [36.5%] vs 1304 [34.4%]). At greater than 1 year to 2 years or less after treatment, men with local treatment continued to experience a higher burden of adverse gastrointestinal (99 [6.5%] vs 73 [2.7%]), sexual (456 [29.8%] vs 208 [7.6%]), and urinary (452 [29.5%] vs 454 [16.6%]) conditions, whereas the difference in pain diminished (515 [33.6%] vs 874 [32.0%]). At greater than 2 years to 5 years or less after treatment, men receiving local treatment continued to experience a higher burden of adverse gastrointestinal (95 [7.8%] vs 56 [4.2%]), sexual (488 [40.1%] vs 175 [13.1%]), and urinary (493 [40.5%] vs 348 [26.0%]) conditions compared with men who received nonlocal treatment.

**Figure.**
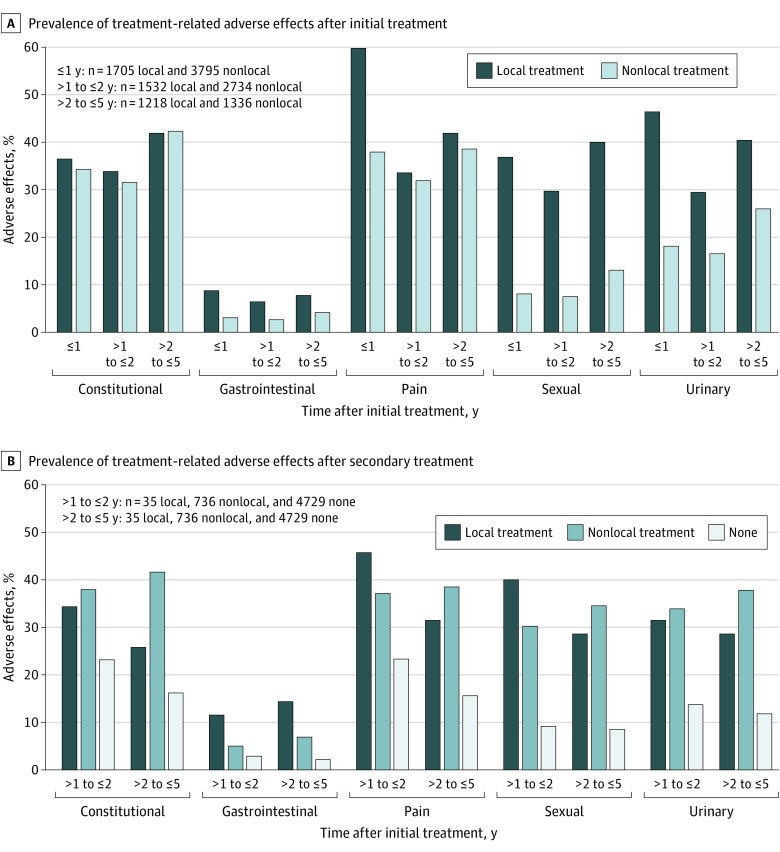
Prevalence of Treatment-Related Adverse Effects After Initial Local or Nonlocal Treatment and Secondary Local or Nonlocal Treatment or No Secondary Treatment at Multiple Time Points

Panel B in the Figure shows the prevalence of the treatment-related adverse effects at 2 time points after secondary treatment as well as among men who received no secondary treatment. At greater than 1 year to 2 years or less after initial treatment, for all conditions, men who received no secondary treatment had a lower prevalence of adverse effects compared with men who received local or nonlocal secondary treatment. Men who received local secondary treatment had a higher prevalence of adverse gastrointestinal (4 [11.4%] vs 36 [4.9%]), pain (16 [45.7%] vs 273 [37.1%]), and sexual (14 [40.0%] vs 222 [30.2%]) conditions compared with men who received nonlocal secondary treatment at greater than 1 year to 2 years or less after initial treatment. The prevalence of constitutional (12 [34.3%] vs 279 [37.9%]) and urinary (11 [31.4%] vs 249 [33.8%]) conditions was similar across the treatment groups. At greater than 2 years to 5 years or less after treatment, men who received local vs nonlocal secondary treatment had a higher burden of adverse gastrointestinal conditions (5 [14.3%] vs 50 [6.8%]).

### Association of Local Treatment With Treatment-Related Adverse Effects

The associations between (1) local treatment vs nonlocal treatment at 1 year or less, (2) initial local treatment with any secondary treatment vs nonlocal initial treatment at greater than 1 year to 2 years or less and greater than 2 years to 5 years or less after initial treatment, and (3) local treatment without secondary treatment vs nonlocal initial treatment at greater than 1 year to 2 years or less and greater than 2 years to 5 years or less after initial treatment were assessed for each condition. The AORs and 95% CIs for each period are given in Table 2.

**Table 2.** Associations Between Treatment Type and Treatment-Related Adverse Effects by Time From Initial Treatment Among 5502 Men Diagnosed With T4N1 M1 Cancer at the Veterans Health Administration From 1997 to 2013

Treatment comparison	Time after initial treatment, y	Adjusted odds ratio (95% CI)^a^	*P* value
**Outcome: constitutional adverse effects**			
Local vs nonlocal treatment only	≤1	1.07 (0.91-1.25)	.41
First local and any second treatment vs nonlocal	>1-≤2	1.50 (1.18-1.91)	<.001
	>2-≤5	1.78 (1.35-2.35)	<.001
First local and no second treatment vs nonlocal treatment only	>1-≤2	0.91 (0.75-1.11)	.36
	>2-≤5	0.83 (0.65-1.04)	.11
**Outcome: gastrointestinal adverse effects**			
Local vs nonlocal treatment only	≤1	4.08 (3.06-5.45)	<.001
First local and any second treatment vs nonlocal	>1-≤2	4.09 (2.47-6.79)	<.001
	>2-≤5	4.01 (2.38-6.75)	<.001
First local and no second treatment vs nonlocal treatment only	>1-≤2	4.00 (2.74-5.84)	<.001
	>2-≤5	2.39 (1.52-3.77)	<.001
**Outcome: pain adverse effects**			
Local vs nonlocal treatment only	≤1	1.57 (1.35-1.83)	<.001
First local and any second treatment vs nonlocal	>1-≤2	1.59 (1.25-2.02)	<.001
	>2-≤5	1.32 (1.01-1.73)	.05
First local and no second treatment vs nonlocal treatment only	>1-≤2	0.95 (0.78-1.15)	.57
	>2-≤5	0.94 (0.74-1.18)	.58
**Outcome: sexual adverse effects**			
Local vs nonlocal treatment only	≤1	2.96 (2.42-3.62)	<.001
First local and any second treatment vs nonlocal	>1-≤2	2.77 (2.06-3.73)	<.001
	>2-≤5	3.59 (2.62-4.92)	<.001
First local and no second treatment vs nonlocal treatment only	>1-≤2	2.94 (2.30-3.77)	<.001
	>2-≤5	3.36 (2.56-4.41)	<.001
**Outcome: urinary adverse effects**			
Local vs nonlocal treatment only	<1	2.25 (1.90-2.66)	<.001
First local and any second treatment vs nonlocal	>1-≤2	2.24 (1.73-2.91)	<.001
	>2-≤5	2.10 (1.58-2.78)	<.001
First local and no second treatment vs nonlocal treatment only	>1-≤2	1.46 (1.18-1.82)	<.001
	>2-≤5	1.39 (1.09-1.78)	.01

^a^ Adjusted for age at diagnosis, race, body mass index, node positivity, stage, grade, location (urban vs rural), academic center (yes vs no), and family history of malignancy.

#### Constitutional Conditions

No association was observed between receipt of local treatment (vs nonlocal treatment) and constitutional conditions at 1 year or less after initial treatment. Local treatment followed by any secondary treatment was associated with a higher likelihood of developing constitutional conditions at greater than 1 year to 2 years or less (AOR, 1.50; 95% CI, 1.18-1.91) and greater than 2 years to 5 years or less (AOR, 1.78; 95% CI, 1.35-2.35) after initial treatment. Local treatment without secondary treatment (vs nonlocal treatment) was not associated with constitutional conditions.

#### Gastrointestinal Conditions

Men who received initial local treatment (vs nonlocal treatment) were more likely to develop gastrointestinal conditions (AOR, 4.08; 95% CI, 3.06-5.45) at less than 1 year after initial treatment. Local treatment with any secondary treatment was also associated with higher odds of gastrointestinal conditions at greater than 1 year to 2 years or less (AOR, 4.09; 95% CI, 2.47-6.79) and greater than 2 years to 5 years or less (AOR, 4.01; 95% CI, 2.38-6.75) after initial treatment. Similarly, men who received local treatment without secondary treatment were significantly more likely to experienced gastrointestinal conditions at both greater than 1 year to 2 years or less and greater than 2 years to 5 years or less after initial treatment (AOR, 2.39; 95% CI, 1.52-3.77).

#### Pain

Men who received initial local treatment (vs nonlocal treatment) were more likely to experience pain (AOR, 1.57; 95% CI, 1.35-1.83) at 1 year or less after initial treatment. Moreover, initial local treatment with secondary treatment was associated with pain at greater than 1 year to 2 years or less (AOR, 1.59; 95% CI, 1.25-2.02) and greater than 2 years to 5 years or less (AOR, 1.32; 95% CI, 1.01-1.73) after initial treatment. No associations were observed between local treatment without secondary treatment at either greater than 1 year to 2 years or less or greater than 2 years to 5 years or less after initial treatment.

#### Sexual Conditions

Men who received initial local treatment (vs nonlocal treatment) were more likely to experience adverse sexual conditions (AOR, 2.96; 95% CI, 2.42-3.62) at 1 year or less after initial treatment. Moreover, men who received local treatment followed by secondary treatment were also more likely to experience adverse sexual conditions at greater than 1 year to 2 years or less (AOR, 2.77; 95% CI, 2.06-3.73) and greater than 2 years to 5 years or less (AOR, 3.59; 95% CI, 2.62-4.92) after initial treatment. Similarly, local treatment without secondary treatment was also significantly associated with adverse sexual conditions at both greater than 1 year to 2 years or less and greater than 2 years to 5 years or less after initial treatment (AOR, 3.36; 95% CI, 2.56-4.41).

#### Urinary Conditions

Local treatment (vs nonlocal treatment) was associated with a higher likelihood of urinary conditions (AOR, 2.25; 95% CI, 1.90-2.66) at 1 year or less after initial treatment. Initial local treatment with secondary treatment was also associated with adverse urinary conditions at greater than 1 year to 2 years or less (AOR, 2.24; 95% CI, 1.73-2.91) and greater than 2 years to 5 years or less (AOR, 2.10; 95% CI, 1.58-2.78) after initial treatment. Similarly, initial local treatment without secondary treatment later was significantly associated with adverse sexual conditions at greater than 1 year to 2 years or less and greater than 2 years to 5 years or less after initial treatment (AOR, 1.39; 95% CI, 1.09-1.78).

### Sensitivity Analyses

Additional adjustment for prostate-specific antigen resulted in similar findings. eTables 2 and 3 in Supplement 1 show the association between treatment type and adverse effects among the subgroups of men receiving RP or radiation as local treatment. Notably, in the RP subgroup, the magnitude of the association of local treatment with sexual adverse effects was higher (AOR, 5.96; 95% CI, 3.37-10.5) at 1 year or less after initial treatment.

eTable 4 in Supplement 1 describes the more restrictive T4M1 cohort. The cohort is similar to the T4N1M1 cohort. eFigure 2 in Supplement 1 displays the prevalence of the adverse effects for initial and secondary treatment for the restricted cohort. Consistent with the primary analytic cohort, gastrointestinal, pain, sexual, and urinary pain adverse conditions were more prevalent in men who received local treatment at all time points. eTable 5 in Supplement 1 displays the AORs and 95% CIs for local treatment (with and without secondary treatment) vs nonlocal initial treatment at 1 year or less, greater than 1 year to 2 years or less, and greater than 2 years to 5 years or less after initial treatment and treatment-related adverse effects (restricted cohort). Results are consistent with the initial analytic cohort.

In another sensitivity analysis, we repeated our analyses restricting the cohorts (T4N1M1 and T4M1) to men who survived a minimum of 5 years after initial treatment. eFigures 3 and 4 in Supplement 1 display the prevalence of the adverse effects for men who survived at least 5 years and were diagnosed with T4N1M1 disease and T4M1 disease. eTables 6 and 7 in Supplement 1 display the AORs and 95% CIs for local treatment (with or without secondary treatment) vs nonlocal treatment at all 3 time points among men who survived at least 5 years and were diagnosed with T4N1M1 disease and T4M1 disease. Findings of these analyses were consistent with previous findings. In general, local treatment was associated with adverse constitutional, gastrointestinal, pain, sexual, and urinary conditions across all 3 time points up to 5 years after initial treatment.

## Discussion

In our nationally representative sample of US veterans diagnosed with advanced PCa, local treatment with RP or radiation was associated with adverse effects across multiple domains. Constitutional and pain symptoms remained associated with local treatment up to 2 years after treatment, whereas gastrointestinal, sexual, and urinary symptoms remained significantly associated with local treatment up to 5 years after treatment. To our knowledge, this is one of the first studies to examine local treatment among men with advanced PCa across multiple adverse conditions up to 5 years after initial treatment. These findings are of increasing clinical importance because there is an increasing incidence of advanced PCa and because therapies for advanced disease continue to rapidly evolve, making consideration of adverse effects even more important.^17,18,19,20^

Our results are consistent with the known side effect profiles in patients with clinically localized PCa receiving surgery or radiation vs active surveillance. The higher rates of treatment-associated morbidity in localized patients are well known. Studies^8,21^ have found that patient-reported bother with sexual dysfunction at 3 to 5 years after treatment is similar between surgery and radiation cohorts but significantly worse compared with surveillance cohorts. Moderate to severe urinary incontinence after prostatectomy appears to be worse than after radiation or surveillance, whereas moderate to severe gastrointestinal symptom bother after prostatectomy appears to be significantly better than after radiation or surveillance.^8,21^ Our results extend these findings to advanced PCa.

The current standard of care for men with advanced PCa is ADT. Thus, the comparison group is not active surveillance, for which expected treatment-related adverse effects should be minimal, but ADT. Like local treatment, ADT is associated with a host of adverse effects.^22^ In our study, 67.1% of men receiving nonlocal treatment experienced adverse conditions across at least 1 domain at greater than 2 years to 5 years or less after initial diagnosis. Although this prevalence is lower than among men receiving local treatment (75.2%), the overall prevalence of adverse effects is high among both treatment groups. However, sexual (40.1% vs 13.1%) and urinary (40.5% vs 26.0%) adverse effects remain higher in men receiving local treatment compared with men receiving nonlocal treatment greater than 2 years to 5 years or less after treatment. Together these findings suggest the importance of considering adverse effects when making informed treatment decisions.

Informed decision-making by both patients and practitioners must account for the potential for enhanced survival vs the potential for prolonged adverse effects and quality of life.^23^ Such decision making is especially important in the context of advanced disease, where the survival benefit of local treatment may not be substantial. Indeed, treatment-related decisional regret among patients with PCa is common when patient expectations and experiences are misaligned.^24,25,26,27^ Our results suggest that patients and clinicians should carefully consider potential treatment-related adverse effects, especially because there are currently no established guidelines regarding the use of local treatment among men with advanced PCa.

### Strengths and Limitations

Our study had several strengths. To our knowledge, this is one of the first studies to examine the association between local treatment and treatment-related adverse effects across multiple domains in men with advanced PCa up to 5 years after treatment. Our cohort consists of a racially diverse (approximately 30% Black) and nationally representative sample of all men diagnosed with advanced PCa during a 14-year period at any Veterans Affairs hospital across the country. Lastly, because Veterans Affairs provides care to all veterans who enrolled in the VHA, our study is able to control for access to care in ways other studies are unable to.

This study also has some limitations. Our study is limited by the retrospective nature of the cohort. Thus, there is a potential for confounding due to unmeasured variables or bias given that receipt of local treatment is influenced by patient and clinician preference. In our cohort, we observed that men who received local treatment were, on average, younger. Thus, it is possible that if older or less healthy patients (eg, with more frailty or comorbidities or less fitness or life expectancy) receive local treatment, they may experience worse adverse effects than observed in our study. If anything, this possibility may have attenuated our findings. However, we still observed significant associations between local treatment and adverse effects. Identification of our outcomes using coding and medications may have also resulted in underreporting of adverse effects, and there could be bias if certain codes are used differentially across treatments. Moreover, coding inaccuracies could have resulted in misclassification. In addition, the VHA patient population may not be generalizable to all patients with PCa. Finally, men who were identified as having advanced disease due to N1 status alone may be clinically distinct from other men with advanced disease. However, in our sensitivity analysis, we obtained similar results when limiting the cohort to men with T4M1 disease only.

## Conclusions

In this cohort study, receipt of local treatment in the setting of advanced PCa was associated with significantly more treatment-related adverse effects across constitutional, gastrointestinal, pain, sexual, and urinary domains up to 5 years after treatment. These results suggest that patients and clinicians should consider the adverse effects of local treatment when making treatment decisions in the setting of advanced PCa.

## Notes

Supplement 1. **eTable 1.** Codes and Medications to Define Outcomes **eTable 2.** Subgroup Surgery: Associations (ORs and 95% CI) Between Treatment Type and Treatment-Related Side Effects by Time From Initial Treatment Among Men Diagnosed With T4, N1, or M1 (N = 5502) Cancer at the Veterans Health Administration Between 1997 and 2013, Excluding Those Who Received Radiation as Local Treatment (N = 762) **eTable 3.** Subgroup Radiation: Associations (ORs and 95% CI) Between Treatment Type and Treatment-Related Side Effects by Time From Initial Treatment Among Men Diagnosed With T4, N1, or M1 Cancer (N = 5502) at the Veterans Health Administration Between 1997 and 2013, Excluding Those Who Received Surgery as Local Treatment (N = 1038) **eTable 4.** Characteristics of Men identified From the VACCR as Being Diagnosed With Prostate Cancer Between the Years 1997 and 2013 –T4 or M1 Cohort **eTable 5.** Associations (ORs and 95% CI) Between Treatment Type and Treatment-Related Side Effects by Time From Initial Treatment Among Men Diagnosed With T4 or M1 Cancer at the Veterans Health Administration Between 1997 and 2013 (N = 3438) **eTable 6.** Associations (ORs and 95% CI) Between Treatment Type and Treatment-Related Side Effects by Time From Initial Treatment Among Men Diagnosed With T4, M1, or N1 Cancer at the Veterans Health Administration Between 1997 and 201—Limited to Men With at Least 5 Years of Follow-Up **eTable 7.** Associations (ORs and 95% CI) Between Treatment Type and Treatment-Related Side Effects by Time From Initial Treatment Among Men Diagnosed With T4 or M1 Cancer at the Veterans Health Administration Between 1997 and 2013—Limited to Men With at Least 5 Years of Follow-Up **eFigure 1.** Flowchart of Study Population—T4/M1/N1 Cohort **eFigure 2.** T4/M1 Cohort—Prevalence of Side Effects **eFigure 3.** T4/N1/M1 Cohort—Cohort Restricted to Men With at Least 5 Years of Follow-Up **eFigure 4.** T4/M1 Cohort—Cohort Restricted to Men With at Least 5 Years of Follow-Up

Supplement 2.
Data Sharing Statement
