# Supplementary material for: Local Treatment and Treatment-Related Adverse Effects Among Patients With Advanced Prostate Cancer
Source: JAMA Netw Open. 2023 Dec 18;6(12):e2348057. doi: 10.1001/jamanetworkopen.2023.48057 (PMC10728764; doi:10.1001/jamanetworkopen.2023.48057)
Supplement: Supplement 1. — eTable 1. Codes and Medications to Define Outcomes eTable 2. Subgroup Surgery: Associations (ORs and 95% CI) Between Treatment Type and Treatment-Related Side Effects by Time From Initial Treatment Among Men Diagnosed With T4, N1, or M1 (N = 5502) Cancer at the Veterans Health Administration Between 1997 and 2013, Excluding Those Who Received Radiation as Local Treatment (N = 762) eTable 3. Subgroup Radiation: Associations (ORs and 95% CI) Between Treatment Type and Treatment-Related Side Effects by Time From Initial Treatment Among Men Diagnosed With T4, N1, or M1 Cancer (N = 5502) at the Veterans Health Administration Between 1997 and 2013, Excluding Those Who Received Surgery as Local Treatment (N = 1038) eTable 4. Characteristics of Men identified From the VACCR as Being Diagnosed With Prostate Cancer Between the Years 1997 and 2013 –T4 or M1 Cohort eTable 5. Associations (ORs and 95% CI) Between Treatment Type and Treatment-Related Side Effects by Time From Initial Treatment Among Men Diagnosed With T4 or M1 Cancer at the Veterans Health Administration Between 1997 and 2013 (N = 3438) eTable 6. Associations (ORs and 95% CI) Between Treatment Type and Treatment-Related Side Effects by Time From Initial Treatment Among Men Diagnosed With T4, M1, or N1 Cancer at the Veterans Health Administration Between 1997 and 201—Limited to Men With at Least 5 Years of Follow-Up eTable 7. Associations (ORs and 95% CI) Between Treatment Type and Treatment-Related Side Effects by Time From Initial Treatment Among Men Diagnosed With T4 or M1 Cancer at the Veterans Health Administration Between 1997 and 2013—Limited to Men With at Least 5 Years of Follow-Up eFigure 1. Flowchart of Study Population—T4/M1/N1 Cohort eFigure 2. T4/M1 Cohort—Prevalence of Side Effects eFigure 3. T4/N1/M1 Cohort—Cohort Restricted to Men With at Least 5 Years of Follow-Up eFigure 4. T4/M1 Cohort—Cohort Restricted to Men With at Least 5 Years of Follow-Up [file jamanetwopen-e2348057-s001.pdf]

Supplementary Online Content

Khan S, Chang S-H, Wang M, et al. Local treatment and treatment-related adverse effects among patients with advanced prostate cancer. *JAMA Netw Open*. 2023;6(12):e2348057. doi:10.1001/jamanetworkopen.2023.48057

eTable 1. Codes and Medications to Define Outcomes

eTable 2. Subgroup Surgery: Associations (ORs and 95% CI) Between Treatment Type and Treatment-Related Side Effects by Time From Initial Treatment Among Men Diagnosed With T4, N1, or M1 (N = 5502) Cancer at the Veterans Health Administration Between 1997 and 2013, Excluding Those Who Received Radiation as Local Treatment (N = 762)

eTable 3. Subgroup Radiation: Associations (ORs and 95% CI) Between Treatment Type and Treatment-Related Side Effects by Time From Initial Treatment Among Men Diagnosed With T4, N1, or M1 Cancer (N = 5502) a the Veterans Health Administration Between 1997 And 2013, Excluding Those Who Received Surgery as Local Treatment (N = 1038)

eTable 4. Characteristics of Men identified From the VACCR as Being Diagnosed With Prostate Cancer Between the Years 1997 and 2013 –T4 or M1 Cohort

eTable 5. Associations (ORs and 95% CI) Between Treatment Type and Treatment-Related Side Effects by Time From Initial Treatment Among Men Diagnosed With T4 or M1 Cancer at the Veterans Health Administration Between 1997 and 2013 (N = 3438)

eTable 6. Associations (ORs and 95% CI) Between Treatment Type and Treatment-Related Side Effects by Time From Initial Treatment Among Men Diagnosed With T4 , M1, or N1 Cancer at the Veterans Health Administration Between 1997 and 2013 –Limited to Men With at Least 5 Years of Follow-Up

eTable 7. Associations (ORs and 95% CI) Between Treatment Type and Treatment-Related Side Effects by Time From Initial Treatment Among Men Diagnosed With T4 or M1 Cancer at the Veterans Health Administration Between 1997 and 2013—Limited to Men With at Least 5 Years of Follow-Up

eFigure 1. Flowchart of Study Population—T4/M1/N1 Cohort

eFigure 2. T4/M1 Cohort—Prevalence of Side Effects

eFigure 3. T4/N1/M1 Cohort—Cohort Restricted to Men with at Least 5 Years of Follow-Up

eFigure 4. T4/M1 Cohort—Cohort Restricted to Men with at Least 5 Years of Follow-Up

This supplementary material has been provided by the authors to give readers additional information about their work.

© 2023 Khan S et al. *JAMA Network Open.*

eTable 1. Codes and Medications to Define Outcomes

Category	Condition	ICD codes	Medications	Surgeries	CPT codes
Urinary	Incontinence	788.3, 788.4, 788.6, 788.9, N39	Diaper, pad, underpands	Artificial urinary sphincter, male sling	53444-53449, 57288
	Cystitis	595, N30	oxybutynin, tolterodine, solifenacin, trospium, fesoterodine, myrbetriq, Botox		
	Urinary overactive bladder	596.51, N32.8	oxybutynin, tolterodine, solifenacin, trospium, fesoterodine, myrbetriq, Botox		
GI	Diarrhea	787.91, R19.7	loperamide, atropine/diphenoxylate		
	Proctitis	558.1, 556.2, 569.49, 987, K62.7			
Sexual	Erectile dysfunction	607.84, N52	Viagra (sildenafil), cialis (tadalafil), levitra (vardenafil), Trimix (alprostadil), Caverject (alprostadil)	Penile prosthesis	54405, 54406, 54408, 54410
Constitu-tional	Hot flashes	627.2, 627.9, N95.1	Megace (megestrol)		
	Depression	296.2, 296.3, 309, 300.4, F32, F33, F34.1, F34.81, F54	citalopram, fluoxetine, sertraline		
	Fatigue	780.8, R53.8, R53.1			
	Anxiety	300.00, 300.01, 300.02, 300.09, F41.1, F41.9	clonazepam, lorazepam, temazepam		
	Poor sleep	V69.4, 780.52, Z72.820, G47.00	trazodone, zolpidem		
Pain	Chronic pain	338.4, G89.4	hydrocodone, morphine, oxycodone, opioid		

© 2023 Khan S et al. *JAMA Network Open.*

eTable 2. Sub-group Surgery: Associations (ORs and 95% CI) between treatment type and treatment-related side effects by time from initial treatment among men diagnosed with T4, N1, or M1 (n=5,502) cancer at the Veterans Health Administration between 1997 and 2013. Excluding those who received radiation as local treatment (n=762)

Treatment comparison	Time after initial treatment	Adjusted OR [a]	Upper CI	Lower CI	p-value
**Outcome: Constitutional Side Effects**					
Local vs. non-local treatment only	≤1 year	0.86	0.53	1.40	0.54
1st local + any 2nd treatment vs. non-local	>1 year ≤ 2 year	1.36	0.94	1.98	0.11
	>2 to ≤ 5 year	1.73	1.21	2.47	0.003
1st local + no 2nd treatment vs. non-local treatment only	>1 year ≤ 2 year	0.62	0.40	0.94	0.02
	>2 to ≤ 5 year	0.71	0.48	1.05	0.08
**Outcome: GI Side Effects**					
Local vs. non-local treatment only	≤ 1 year	2.17	2.14	2.20	<0.001
1st local + any 2nd treatment vs. non-local	>1 year ≤ 2 year	1.67	0.50	5.57	0.95
	>2 to ≤ 5 year	1.43	0.64	3.12	0.38
1st local + no 2nd treatment vs. non-local treatment only	>1 year ≤ 2 year	1.16	0.33	4.11	0.41
	>2 to ≤ 5 year	0.70	0.28	1.80	0.47
**Outcome: Pain Side Effects**					
Local vs. non-local treatment only	≤1 year	1.96	1.22	3.14	0.01
1st local + any 2nd treatment vs. non-local	>1 year ≤ 2 year	1.50	1.02	2.21	0.04
	>2 to ≤ 5 year	1.14	0.80	1.62	0.47
1st local + no 2nd treatment vs. non-local treatment only	>1 year ≤ 2 year	0.84	0.55	1.29	0.42
	>2 to ≤ 5 year	0.83	0.57	1.22	0.34
**Outcome: Sexual Side Effects**					
Local vs. non-local treatment only	≤ 1 year	5.96	3.37	10.5	<0.001
	>1 year ≤ 2 year	3.27	2.12	5.05	<0.001
1st local + any 2nd treatment vs. non-local	>2 to ≤ 5 year	3.92	2.59	6.00	<0.001

© 2023 Khan S et al. *JAMA Network Open.*

1st local + no 2nd treatment vs. non-local treatment only	>1 year ≤ 2 year	4.52	2.89	7.07	<0.001
	>2 to ≤ 5 year	3.72	2.41	5.74	<0.001
**Outcome: Urinary Side Effects**					
Local vs. non-local treatment only	≤1 year	6.24	3.84	10.2	<0.001
1st local + any 2nd treatment vs. non-local	>1 year ≤ 2 year	3.46	2.27	5.29	<0.001
	>2 to ≤ 5 year	2.11	1.45	3.06	<0.001
1st local + no 2nd treatment vs. non-local treatment only	>1 year ≤ 2 year	2.39	1.52	3.76	<0.001
	>2 to ≤ 5 year	1.46	0.98	2.17	0.06

[a] Adjusted for age at diagnosis, race, BMI, node positivity, stage, grade, location (urban vs. rural), academic center (yes vs. no), family history of reportable malignancy

© 2023 Khan S et al. *JAMA Network Open.*

eTable 3. Sub-group Radiation: Associations (ORs and 95% CI) between treatment type and treatment-related side effects by time from initial treatment among men diagnosed with T4, N1, or M1 cancer (n=5,502) at the Veterans Health Administration between 1997 and 2013. Excluding those who received surgery as local treatment (n=1038).

Treatment comparison	Time after initial treatment	Adjusted OR [a]	Upper CI	Lower CI	p-value
**Outcome: Constitutional Side Effects**					
Local vs. non-local treatment only	≤1 year	1.18	0.96	1.47	0.12
1st local + any 2nd treatment vs. non-local	>1 year ≤ 2 year	1.39	0.79	2.45	0.26
	>2 to ≤ 5 year	1.72	0.99	2.99	0.06
1st local + no 2nd treatment vs. non-local treatment only	>1 year ≤ 2 year	0.98	0.73	1.32	0.89
	>2 to ≤ 5 year	0.88	0.66	1.16	0.35
**Outcome: GI Side Effects**					
Local vs. non-local treatment only	≤1 year	4.58	3.15	6.66	<0.001
1st local + any 2nd treatment vs. non-local	>1 year ≤ 2 year	11.42	4.95	26.36	<0.001
	>2 to ≤ 5 year	6.83	3.26	14.29	<0.001
1st local + no 2nd treatment vs. non-local treatment only	>1 year ≤ 2 year	5.83	3.27	10.37	<0.001
	>2 to ≤ 5 year	3.01	1.85	4.89	<0.001
**Outcome: Pain Side Effects**					
Local vs. non-local treatment only	≤1 year	1.35	1.10	1.68	0.01
1st local + any 2nd treatment vs. non-local	>1 year ≤ 2 year	2.27	1.29	4.01	0.005
	>2 to ≤ 5 year	1.59	0.91	2.79	0.10
1st local + no 2nd treatment vs. non-local treatment only	>1 year ≤ 2 year	0.86	0.62	1.20	0.38
	>2 to ≤ 5 year	0.97	0.73	1.28	0.82
**Outcome: Sexual Side Effects**					
Local vs. non-local treatment only	≤1 year	1.90	1.38	2.62	<0.001
1st local + any 2nd treatment vs. non-local	>1 year ≤ 2 year	3.10	1.58	6.06	0.001
	>2 to ≤ 5 year	3.15	1.68	5.92	<0.001
1st local + no 2nd treatment vs. non-local treatment only	>1 year ≤ 2 year	2.30	1.60	3.31	<0.001
	>2 to ≤ 5 year	3.11	2.23	4.33	<0.001

© 2023 Khan S et al. *JAMA Network Open.*

Outcome: Urinary Side Effects					
Local vs. non-local treatment only	≤1 year	1.33	1.03	1.71	0.02
1st local + any 2nd treatment vs. non-local	>1 year ≤ 2 year	2.65	1.40	5.02	0.01
	>2 to ≤ 5 year	2.86	1.63	5.03	<0.001
1st local + no 2nd treatment vs. non-local treatment only	>1 year ≤ 2 year	1.34	0.92	1.94	0.12
	>2 to ≤ 5 year	1.24	0.92	1.68	0.16

[a] Adjusted for age at diagnosis, race, BMI, node positivity, stage, grade, location (urban vs. rural), academic center (yes vs. no), family history of reportable malignancy

© 2023 Khan S et al. *JAMA Network Open.*

eTable 4. Characteristics of Men identified from the VACCR as being diagnosed with prostate cancer between the years 1997 and 2013 –T4 or M1 Cohort

	Metastatic Cancer: T4 or M1					
	Non-local treatment		Local treatment		Total	
N	2,756		682		3,438	
%	80.2		19.8		100.0	
	N	%	N	%	N	%
**Age**						
<50	23	0.8	19	2.8	42	1.2
50-<60	331	12.0	174	25.5	505	14.7
60-<70	765	27.8	299	43.8	1,064	30.9
70+	1,637	59.4	190	27.9	1,827	53.1
**Race** [a]						
Black	828	30.0	217	31.8	1,045	30.4
White	1,854	67.3	440	64.5	2,294	66.7
Other [b]	12	0.4	3	0.4	15	0.4
Unknown	62	2.3	22	3.2	84	2.4
**BMI (kg/m2)**						
<18.5	89	3.2	11	1.6	100	2.9
18.5-<25	985	35.7	185	27.1	1,170	34.0
25-<30	1,021	37.1	269	39.4	1,290	37.5
30+	661	24.0	217	31.8	878	25.5
**Node positivity**						
Yes	502	18.2	81	11.9	583	17.0
No	2,254	81.8	601	88.1	2,855	83.0
**Stage**						
Missing	41	1.5	53	7.8	94	2.7
1	1	0.0	1	0.2	2	0.1
2	15	0.5	161	23.6	176	5.1
3	2	0.1	1	0.2	3	0.1
4	2,697	97.9	466	68.3	3,163	92.0
**Grade**						
Missing	438	15.9	81	11.9	519	15.1
1	17	0.6	4	0.6	21	0.6
2	352	12.8	141	20.7	493	14.3
3	1,841	66.8	437	64.1	2,278	66.3
4	108	3.9	19	2.8	127	3.7
**Location**						
Urban	2,021	73.3	498	73.0	2,519	73.3
Rural	664	24.1	172	25.2	836	24.3
Unknown	71	2.6	12	1.8	83	2.4
**Academic**						
Academic	763	27.7	228	33.4	991	28.8
Non-academic	565	20.5	122	17.9	687	20.0
Unknown	1,428	51.8	332	48.7	1,760	51.2
**Family history of any reportable malignancy**						
Yes	702	25.5	248	36.4	950	27.6
No	1,119	40.6	270	39.6	1,389	40.4

© 2023 Khan S et al. *JAMA Network Open.*

Unknown	935	33.9	164	24.1	1099	32.0
**Treatment Type**						
Radical Prostatectomy	0	0.0	238	34.9	238	6.9
Radiation	0	0.0	423	62.0	423	12.3
Adjuvant Surgery and Radiation [c]	0	0.0	21	3.1	21	0.6
Hormone Only	2,696	97.8	0	0.0	2,696	78.4
Chemotherapy	60	2.2	0	0.0	60	1.7

[a] Race was determined from medical records.
[b] Other included Asian, Native American, and Pacific Islander
[c] Defined as adjuvant if within 1 year of initial treatment

© 2023 Khan S et al. *JAMA Network Open.*

eTable 5. Associations (ORs and 95% CI) between treatment type and treatment-related side effects by time from initial treatment among men diagnosed with T4 or M1 cancer at the Veterans Health Administration between 1997 and 2013 (n=3,438)

Treatment comparison	Time after initial treatment	Adjusted OR [a]	Upper CI	Lower CI	p-value
**Outcome: Constitutional Side Effects**					
Local vs. non-local treatment only	≤1 year	1.14	0.93	1.39	0.21
1st local + any 2nd treatment vs. non-local	>1 year ≤ 2 year	1.72	1.15	2.56	0.01
	>2 to ≤ 5 year	2.01	1.23	3.29	0.01
1st local + no 2nd treatment vs. non-local treatment only	>1 year ≤ 2 year	1.05	0.82	1.36	0.69
	>2 to ≤ 5 year	0.82	0.58	1.14	0.23
**Outcome: GI Side Effects**					
Local vs. non-local treatment only	≤1 year	4.16	2.88	6.01	<0.001
1st local + any 2nd treatment vs. non-local	>1 year ≤ 2 year	4.71	2.07	10.70	<0.001
	>2 to ≤ 5 year	3.40	1.38	8.36	0.01
1st local + no 2nd treatment vs. non-local treatment only	>1 year ≤ 2 year	4.92	2.98	8.12	<0.001
	>2 to ≤ 5 year	2.25	1.16	4.37	0.02
**Outcome: Pain Side Effects**					
Local vs. non-local treatment only	≤1 year	1.46	1.20	1.78	<0.001
1st local + any 2nd treatment vs. non-local	>1 year ≤ 2 year	1.67	1.11	2.50	0.01
	>2 to ≤ 5 year	1.55	0.95	2.52	0.08
1st local + no 2nd treatment vs. non-local treatment only	>1 year ≤ 2 year	1.06	0.82	1.37	0.65
	>2 to ≤ 5 year	0.93	0.67	1.31	0.69
**Outcome: Sexual Side Effects**					
Local vs. non-local treatment only	≤1 year	2.45	1.86	3.23	<0.001
1st local + any 2nd treatment vs. non-local	>1 year ≤ 2 year	2.06	1.23	3.44	0.01
	>2 to ≤ 5 year	2.14	1.22	3.77	0.01
1st local + no 2nd treatment vs. non-local treatment only	>1 year ≤ 2 year	2.59	1.84	3.63	<0.001
	>2 to ≤ 5 year	2.56	1.71	3.84	<0.001

© 2023 Khan S et al. *JAMA Network Open.*

Outcome: Urinary Side Effects					
Local vs. non-local treatment only	≤1 year	1.76	1.41	2.20	<0.001
1st local + any 2nd treatment vs. non-local	>1 year ≤ 2 year	2.28	1.49	3.50	<0.001
	>2 to ≤ 5 year	2.16	1.30	3.59	<0.001
1st local + no 2nd treatment vs. non-local treatment only	>1 year ≤ 2 year	1.32	0.99	1.78	0.06
	>2 to ≤ 5 year	1.46	1.01	2.09	0.04

[a] Adjusted for age at diagnosis, race, BMI, node positivity, stage, grade, location (urban vs. rural), academic center (yes vs. no), and family history of reportable malignancy

© 2023 Khan S et al. *JAMA Network Open.*

eTable 6. Associations (ORs and 95% CI) between treatment type and treatment-related side effects by time from initial treatment among men diagnosed with T4 , M1, or N1 cancer at the Veterans Health Administration between 1997 and 2013 –limited to men with at least 5 years of follow-up

Treatment comparison	Time after initial treatment	Adjusted OR [a]	Upper CI	Lower CI	p-value
**Outcome: Constitutional Side Effects**					
Local vs. non-local treatment only	≤1 year	1.07	0.86	1.34	0.55
1st local + any 2nd treatment vs. non-local	>1 year ≤ 2 year	1.52	1.14	2.03	<0.001
	>2 to ≤ 5 year	1.78	1.35	2.35	<0.001
1st local + no 2nd treatment vs. non-local treatment only	>1 year ≤ 2 year	0.86	0.67	1.10	0.23
	>2 to ≤ 5 year	0.83	0.65	1.04	0.11
**Outcome: GI Side Effects**					
Local vs. non-local treatment only	≤1 year	4.39	2.77	6.96	<0.001
1st local + any 2nd treatment vs. non-local	>1 year ≤ 2 year	4.51	2.28	8.95	<0.001
	>2 to ≤ 5 year	4.01	2.38	6.75	<0.001
1st local + no 2nd treatment vs. non-local treatment only	>1 year ≤ 2 year	4.43	2.52	7.76	<0.001
	>2 to ≤ 5 year	2.39	1.52	3.77	<0.001
**Outcome: Pain Side Effects**					
Local vs. non-local treatment only	≤1 year	2.24	1.80	2.79	<0.001
1st local + any 2nd treatment vs. non-local	>1 year ≤ 2 year	1.68	1.25	2.27	<0.001
	>2 to ≤ 5 year	1.32	1.01	1.73	0.05
1st local + no 2nd treatment vs. non-local treatment only	>1 year ≤ 2 year	0.94	0.72	1.22	0.63
	>2 to ≤ 5 year	0.94	0.74	1.18	0.58
**Outcome: Sexual Side Effects**					
Local vs. non-local treatment only	≤1 year	3.24	2.48	4.23	<0.001
1st local + any 2nd treatment vs. non-local	>1 year ≤ 2 year	2.60	1.86	3.62	<0.001
	>2 to ≤ 5 year	3.59	2.62	4.92	<0.001
	>1 year ≤ 2 year	3.00	2.25	4.01	<0.001

© 2023 Khan S et al. *JAMA Network Open.*

1st local + no 2nd treatment vs. non-local treatment only	>2 to ≤ 5 year	3.36	2.56	4.41	<0.001
**Outcome: Urinary Side Effects**					
Local vs. non-local treatment only	≤1 year	3.46	2.70	4.43	<0.001
1st local + any 2nd treatment vs. non-local	>1 year ≤ 2 year	2.79	2.02	3.85	<0.001
	>2 to ≤ 5 year	2.10	1.58	2.78	<0.001
1st local + no 2nd treatment vs. non-local treatment only	>1 year ≤ 2 year	1.79	1.34	2.38	<0.001
	>2 to ≤ 5 year	1.39	1.09	1.78	0.01

[a] Adjusted for age at diagnosis, race, BMI, node positivity, stage, grade, location (urban vs. rural), academic center (yes vs. no), and family history of reportable malignancy

© 2023 Khan S et al. *JAMA Network Open.*

eTable 7. Associations (ORs and 95% CI) between treatment type and treatment-related side effects by time from initial treatment among men diagnosed with T4 or M1 cancer at the Veterans Health Administration between 1997 and 2013 –limited to men with at least 5 years of follow-up

Treatment comparison	Time after initial treatment	Adjusted OR [a]	Upper CI	Lower CI	p-value
**Outcome: Constitutional Side Effects**					
Local vs. non-local treatment only	≤1 year	1.07	0.77	1.49	0.68
1st local + any 2nd treatment vs. non-local	>1 year ≤ 2 year	2.07	1.25	3.44	<0.001
	>2 to ≤ 5 year	2.01	1.23	3.29	0.01
1st local + no 2nd treatment vs. non-local treatment only	>1 year ≤ 2 year	1.02	0.71	1.46	0.92
	>2 to ≤ 5 year	0.82	0.58	1.14	0.23
**Outcome: GI Side Effects**					
Local vs. non-local treatment only	≤1 year	4.40	2.20	8.80	<0.001
1st local + any 2nd treatment vs. non-local	>1 year ≤ 2 year	3.90	1.12	13.65	0.03
	>2 to ≤ 5 year	3.40	1.38	8.36	0.01
1st local + no 2nd treatment vs. non-local treatment only	>1 year ≤ 2 year	4.51	2.02	10.08	<0.001
	>2 to ≤ 5 year	2.25	1.16	4.37	0.02
**Outcome: Pain Side Effects**					
Local vs. non-local treatment only	≤1 year	1.79	1.29	2.46	<0.001
1st local + any 2nd treatment vs. non-local	>1 year ≤ 2 year	1.60	0.94	2.74	0.08
	>2 to ≤ 5 year	1.55	0.95	2.52	0.08
1st local + no 2nd treatment vs. non-local treatment only	>1 year ≤ 2 year	0.90	0.61	1.32	0.58
	>2 to ≤ 5 year	0.93	0.67	1.31	0.69
**Outcome: Sexual Side Effects**					
Local vs. non-local treatment only	≤1 year	2.75	1.86	4.09	<0.001
1st local + any 2nd treatment vs. non-local	>1 year ≤ 2 year	1.73	0.96	3.11	0.07
	>2 to ≤ 5 year	2.14	1.22	3.77	0.01
	>1 year ≤ 2 year	2.61	1.73	3.96	<0.001

© 2023 Khan S et al. *JAMA Network Open.*

1st local + no 2nd treatment vs. non-local treatment only	>2 to ≤ 5 year	2.56	1.71	3.84	<0.001
**Outcome: Urinary Side Effects**					
Local vs. non-local treatment only	≤1 year	2.57	1.79	3.70	<0.001
1st local + any 2nd treatment vs. non-local	>1 year ≤ 2 year	3.90	2.24	6.81	<0.001
	>2 to ≤ 5 year	2.16	1.30	3.59	<0.001
1st local + no 2nd treatment vs. non-local treatment only	>1 year ≤ 2 year	1.72	1.12	2.62	0.01
	>2 to ≤ 5 year	1.46	1.01	2.09	0.04

[a] Adjusted for age at diagnosis, race, BMI, node positivity, stage, grade, location (urban vs. rural), academic center (yes vs. no), and family history of reportable malignancy

© 2023 Khan S et al. *JAMA Network Open.*

eFigure 1. Flow chart of study population - T4/M1/N1 cohort

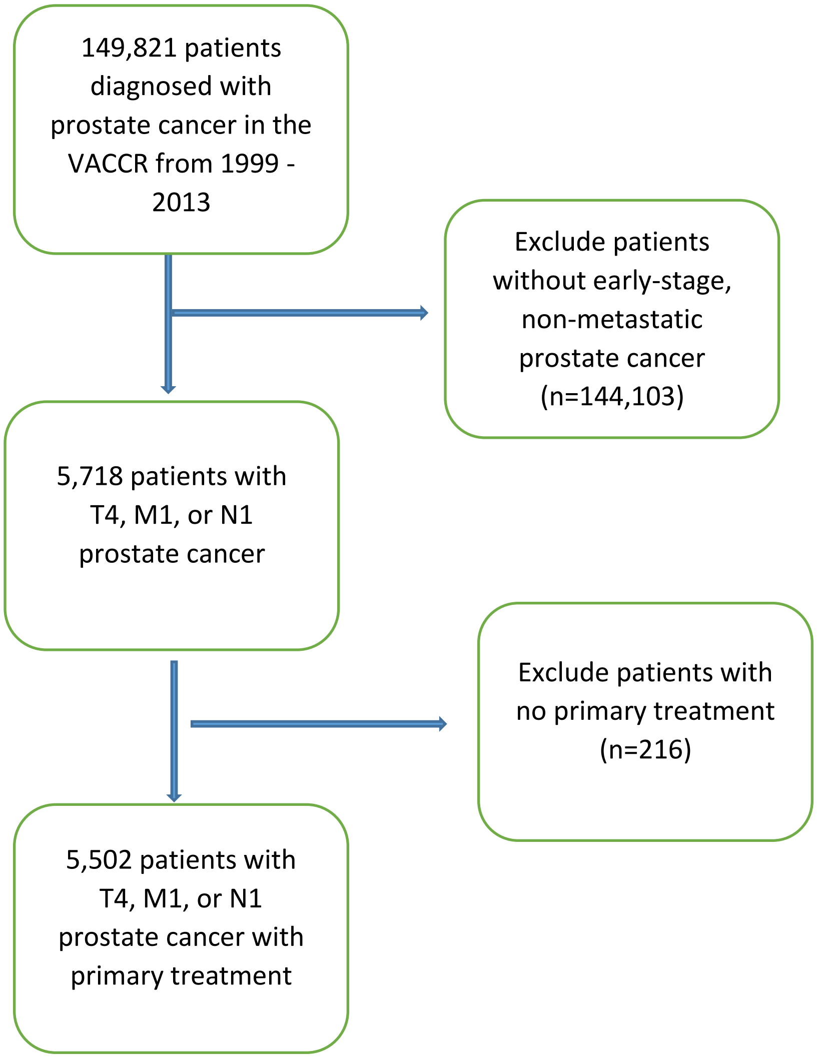

© 2023 Khan S et al. *JAMA Network Open.*

eFigure 2: T4/M1 cohort - prevalence of side effects

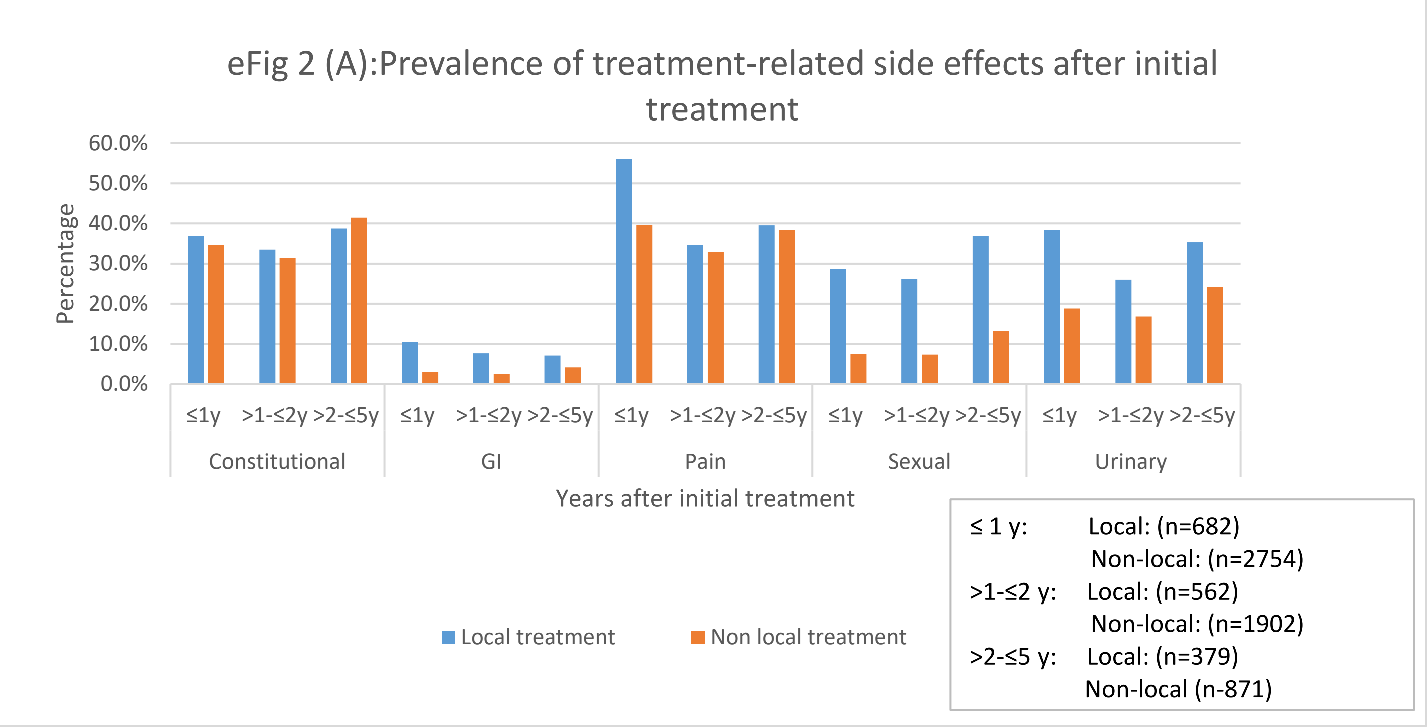

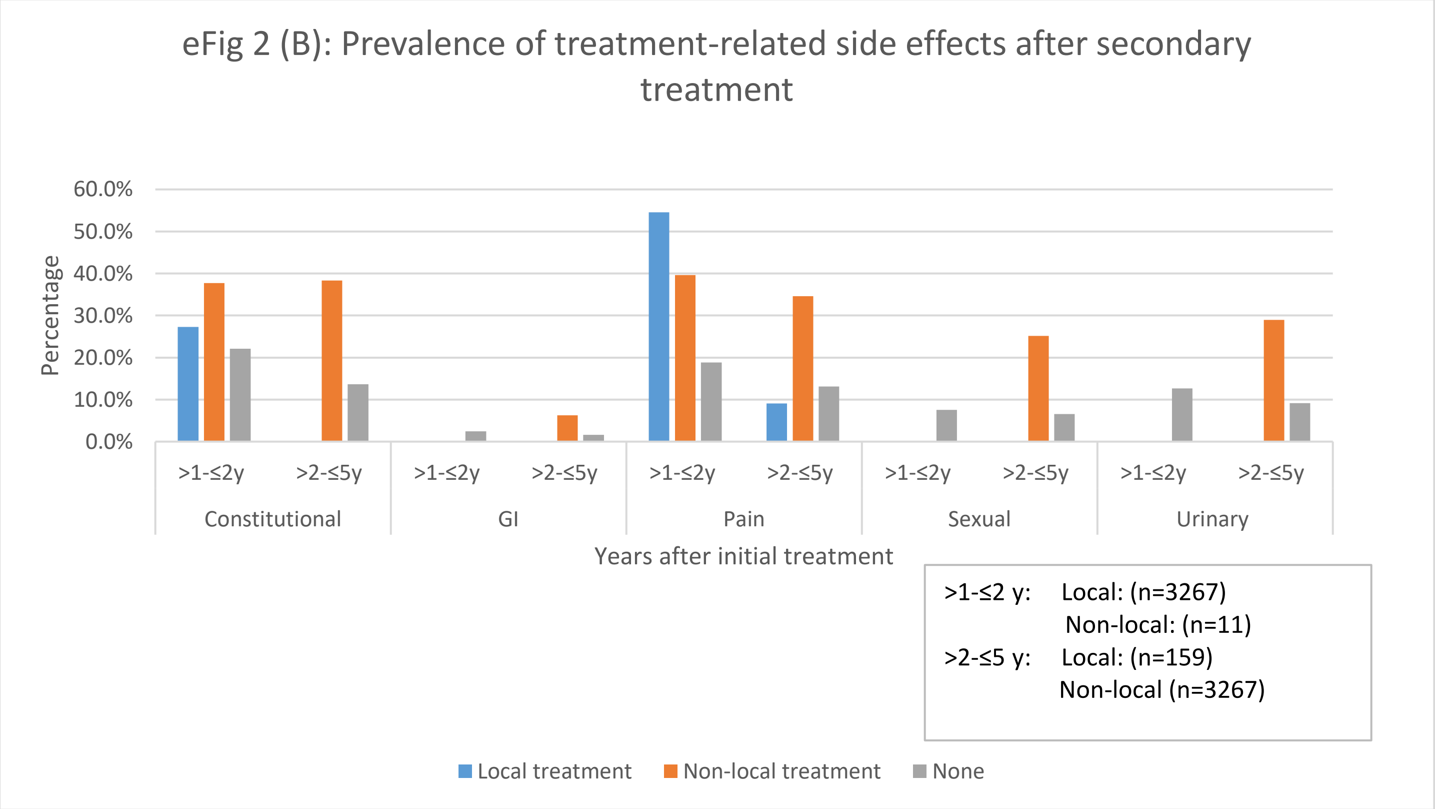

© 2023 Khan S et al. *JAMA Network Open.*

eFigure 3 - T4/N1/M1 – cohort restricted to men with at least 5 years of follow-up

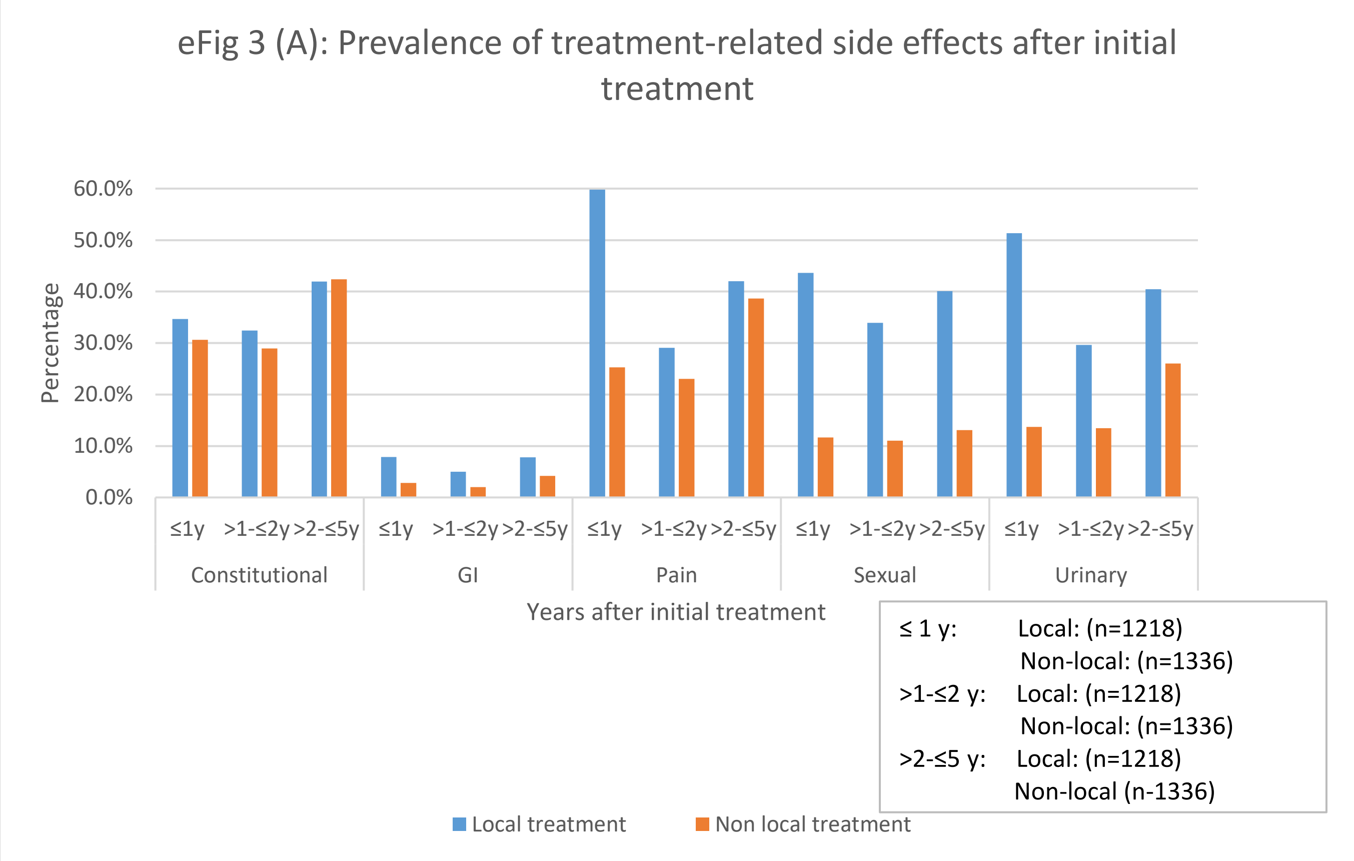

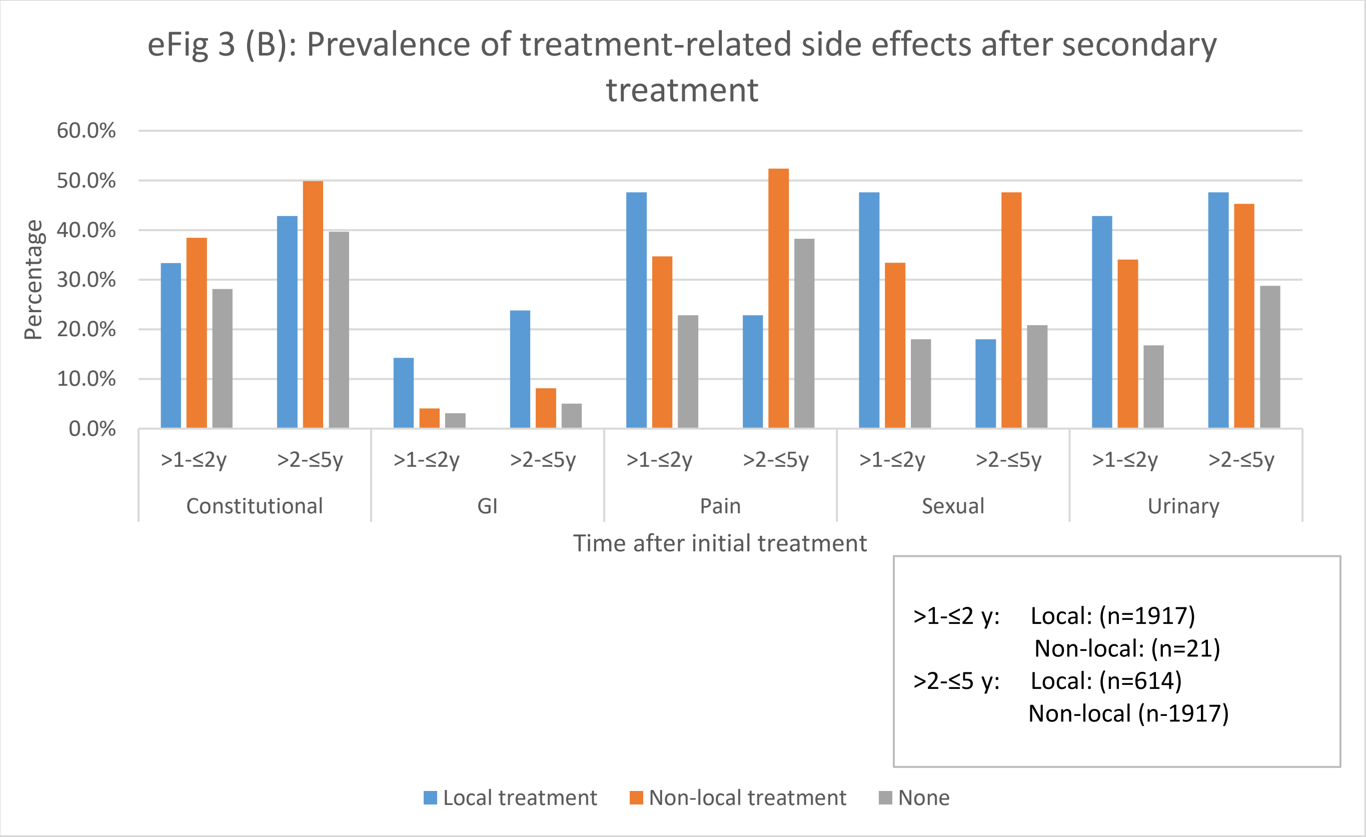

© 2023 Khan S et al. *JAMA Network Open.*

eFigure 4- T4/M1 – restricted to men with at least 5 years of follow-up

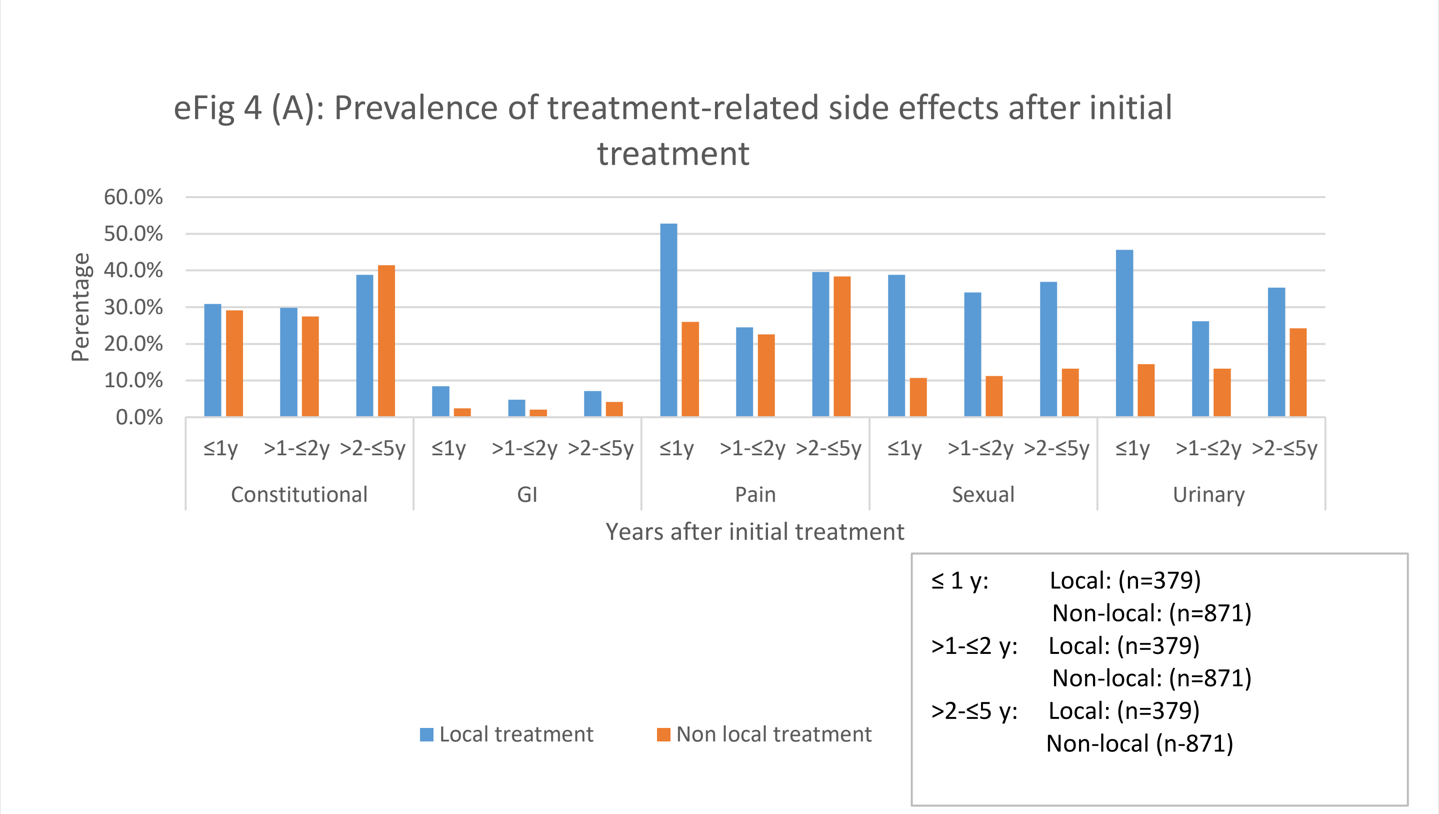

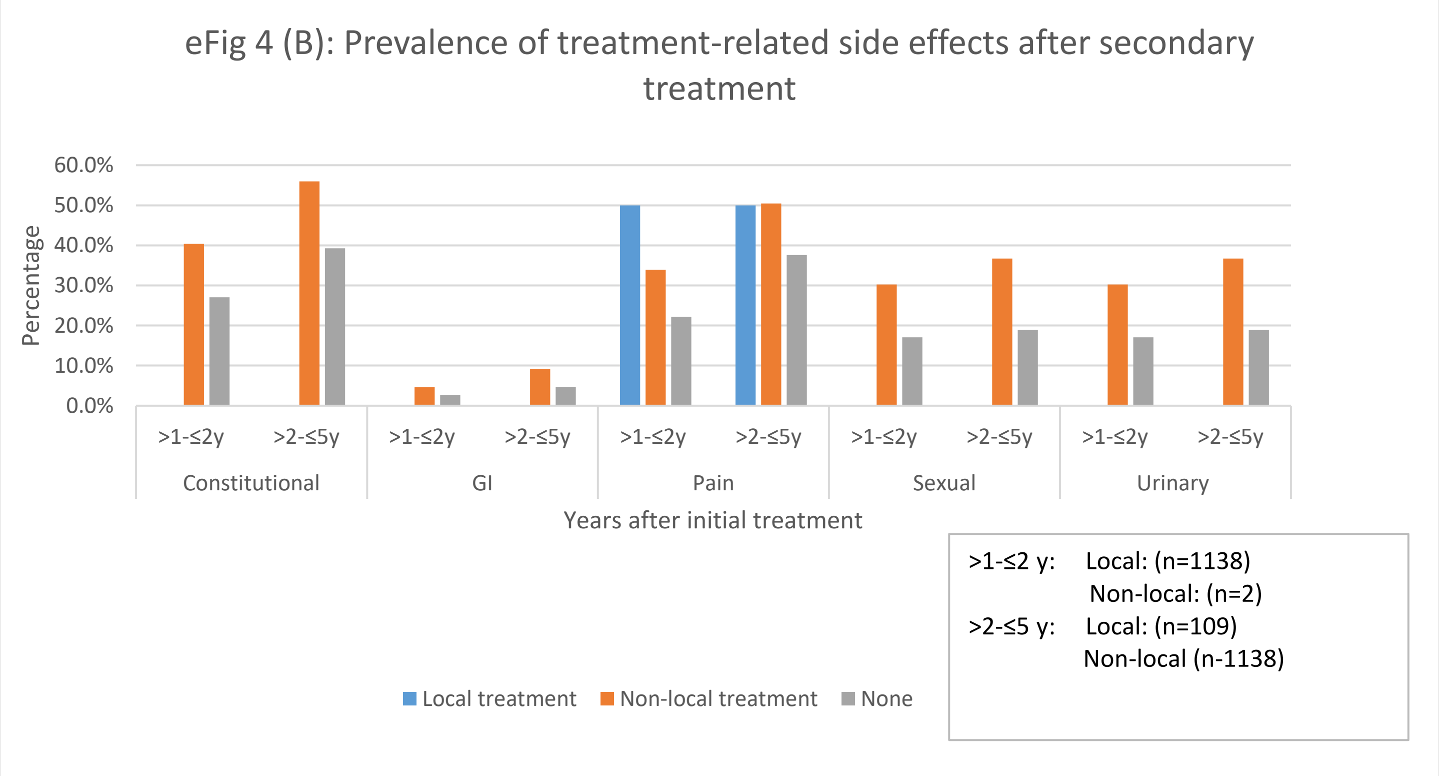

© 2023 Khan S et al. *JAMA Network Open.*
