# Supplementary material for: Local Treatment and Treatment-Related Adverse Effects Among Patients With Advanced Prostate Cancer
Source: JAMA Netw Open. 2023 Dec 18;6(12):e2348057. doi: 10.1001/jamanetworkopen.2023.48057 (PMC10728764; doi:10.1001/jamanetworkopen.2023.48057)
Supplement: Supplement 2. — Data Sharing Statement [file jamanetwopen-e2348057-s002.pdf]

Data Sharing Statement

Khan. Local Treatment and Treatment-Related Adverse Effects Among Patients With Advanced Prostate Cancer. *JAMA Netw Open*. Published December 18, 2023. doi:10.1001/jamanetworkopen.2023.48057

Data
Data available: No

Additional Information
Explanation for why data not available: Access to VA data is governed by the VA and requires a Without Compensation (WOC) appointment through the VA.
